# Our New York Letter

**Published:** 1889-09

**Authors:** M. B. Hutchins

**Affiliations:** New York


### OUR NEW YORK LETTER.

New York, August, 1889.

The other day I asked a well posted physician his method of treating typhoid fever. He replied "expecting" and "stimulating,"—together with the interval administration of naphthalinum "purissimum." The dose is from five to fifteen grains, and is perfectly safe—if pure—having only the unpleasant quality of producing disagreeable eructations. Considers it *the remedy* in typhoid fever, having antiseptic and germicidal properties. One of the best remedies in the enteric diseases, especially those of chronic character in children. Remedy is also useful in fermentative variety of dyspepsia. Give to children with diarrhœa or dysentery, in combination with subnitrate of bismuth.

Antipyrine, like most of our newer remedies, has had a trial in nearly everything. Considered really beneficial in dysmenorrhœa and also in whooping cough. One gentleman mentions the fine effect in the case of a lady—pregnant—and suffering from violent paroxysms of whooping cough—the drug giving relief after the failure of all the usual "remedies." Two physicians, in comparing experience, agreed regarding use of antipyrin, in febrile conditions of children, that there was danger of sudden collapse and death in cases complicated with lung troubles, the foundation of one gentleman's belief being in a case occurring in his own practice. Considered sulphate of quinine per rectum as best antipyretic for chlldren.

A surgeon who is a thorough follower of antiseptic rules gives the following dictations for treatment of sponges, drainage tubes and cat-gut. Those regarding sponges might be considered a curiosity in directions. "(1) Beat free from hard and calcareous particles. (2) Put for fifteen minutes in dilute muriatic acid to dissolve out remnant of lime. (3) Knead by hand, with green soap, in hot water five or ten minutes. (4) Rinse in cold, clear water. (5) Put for twelve hours in two per cent. solution permanganate of potash. (6) Squeeze and put for twelve hours in one per cent. solution of hyposulphite of soda, to which has been added ⅓—½ ounce muriatic acid. (7.) Rinse and put in five per cent. solution of carbolic acid." By this time every germ should have departed from existence!

"Drainage tubes. Put for twenty-four hours in simple cold water, changing once. Keep in five per cent. carbolic acid."

"Cat-gut. (1) Hands being thoroughly disinfected, put the ligatures on glass spools. (2) Put for twelve hours in bichloride 1–1000. (3) Keep in absolute alcohol."

One sees all kinds of aseptic and antiseptic measures employed by the surgeons. And in almost any operation one may see some slight violation of antiseptic rules, perhaps scarcely noticeable, yet, according to the theory, it may be held responsible for the suppuration, in spite of the room having been thoroughly washed in bichloride 1—1000, boiling, "scouring" and placing of instruments in carbolic solution; in spite of following the above directions in preparing the sponges, tubes and ligatures, the wound may suppurate. Why it did is one of the questions which may seem incapable of solution.

### DERMATOLOGY.

A young woman in the skin and cancer hospital had extensive lupus erythematosus, involving face, ears, scalp and forehead, with a few patches on neck and chest. Patches red, scaly, depressed in the center, edges elevated and infiltrated, process being a "round celled" infiltration, the center of which rapidly undergoes atrophic depression. After being for several months under varied external and internal treatment, the case received the following treatment, ordered by Dr. G. T. Elliot, Dr. Bulkley's assistant. On left side of face and nose, application of hydronaphthol plaster, 20 per cent. strength. On right side the new "reducing agent" hydroxylamin (chemically obtained from nitrous acid), beginning with one per cent. strength in alcohol and glycerine, equal parts, used as lotion and let "dry on." Effect of each of the two treatments was to produce an acute local dermatitis. When this occurred, treatment was temporarily suspended until subsidence of inflammation, when the lotion and plaster were again applied upon their respective side of face. On the left side the hydronaphthol plaster was reduced to the 10 per cent. strength, while the hydroxylamin lotion was gradually increased to 3 per cent. strength, which produced very acute inflammation with œdema of lower lid. Lotio ichthyol 3 per cent. applied until process had subsided. At present, six weeks after beginning of treatment, the side receiving the hydroxylamin shows no lupus erythematosus, only patches of irritation covered in places with dried serous discharge. Upon removal of plaster from left side, the face is seen to be perfectly smooth save a few faint scars. The disease itself is always remembered by the patient through the scars left. But it seems that the scars, even, in this case, will hardly be noticeable. No internal, constitutional treatment was given.

Another case of the disease was treated as follows. A 4 per cent. solution of cocaine was first injected under lesions, and they were scraped out with a large curette. Gr. 20 of acid pyrogallic to ℥1 of simple ointment was then applied and produced suppuration of exposed tissues, but seemingly having no effect upon portions of edges which were incompletely curetted. This was followed with application of emplastrum hydrarg. After healing, scaliness of edges persisted, and this was nearly entirely removed by use of the 10 per cent. hydronaphthol plaster, leaving portion apparently free from disease. Other lesions (of nose) which had been curetted received this treatment at once with excellent result. It would seem from the above that this obstinate disease, lupus erythematosus, had found its cure in the hydronaphthol plaster.

A case of chronic psoriasis recently seen, received the following treatment: Upon diffuse much thickened portions, as at knees and elbows, "acid liniment" (made of the following ingredients: ℞—chloroform, 10; chrysarobin, 3; ac. pyrogallic, 3; ac.

salicylic, 3; oil cade, 50; alcohol, 100. *M.*) was applied and the ungt. hg. ammoniat. on the lesions of gyrate, mummular, circinate and annular forms, after patient had received an alkaline bath. Within a week, scaling and thickening wonderfully reduced, but especially well where the acid liniment was applied. Thickening under this treatment declines very fast.

I hear that Dr. Bulkley has given up the standard chrysarobin or pyrogallic treatment in psoriasis, and praises the white precipitate ointment. Dr. Fox says it formerly acted well on cases involving the face and neck, but, perhaps from impurity of the drug, effect does not now seem so good. Dr. F. mentions a case of lichen planus, in a man of very nervous temperament, cured by simply stopping his beer, etc., and putting him on a diet of milk and bread alone. There was no local treatment.

M. B. HUTCHINS, M. D.

